# Silhouette images enable estimation of body fat distribution and associated cardiometabolic risk

**DOI:** 10.1038/s41746-022-00654-1

**Published:** 2022-07-27

**Authors:** Marcus D. R. Klarqvist, Saaket Agrawal, Nathaniel Diamant, Patrick T. Ellinor, Anthony Philippakis, Kenney Ng, Puneet Batra, Amit V. Khera

**Affiliations:** 1 Data Sciences Platform, Broad Institute of MIT and Harvard, Cambridge, MA USA; 2 Cardiovascular Disease Initiative, Broad Institute of MIT and Harvard, Cambridge, MA USA; 3 Center for Genomic Medicine, Department of Medicine, Massachusetts General Hospital, Boston, MA USA; 4 Department of Medicine, Harvard Medical School, Boston, MA USA; 5 Eric and Wendy Schmidt Center, Broad Institute of MIT and Harvard, Cambridge, MA USA; 6 Center for Computational Health, IBM Research, Cambridge, MA USA; 7 Verve Therapeutics, Cambridge, MA USA

**Keywords:** Obesity, Computer science, Medical imaging

## Abstract

Inter-individual variation in fat distribution is increasingly recognized as clinically important but is not routinely assessed in clinical practice, in part because medical imaging has not been practical to deploy at scale for this task. Here, we report a deep learning model trained on an individual’s body shape outline—or “silhouette” —that enables accurate estimation of specific fat depots of interest, including visceral (VAT), abdominal subcutaneous (ASAT), and gluteofemoral (GFAT) adipose tissue volumes, and VAT/ASAT ratio. Two-dimensional coronal and sagittal silhouettes are constructed from whole-body magnetic resonance images in 40,032 participants of the UK Biobank and used as inputs for a convolutional neural network to predict each of these quantities. Mean age of the study participants is 65 years and 51% are female. A cross-validated deep learning model trained on silhouettes enables accurate estimation of VAT, ASAT, and GFAT volumes (*R*^2^: 0.88, 0.93, and 0.93, respectively), outperforming a comparator model combining anthropometric and bioimpedance measures (Δ*R*^2^ = 0.05–0.13). Next, we study VAT/ASAT ratio, a nearly body-mass index (BMI)—and waist circumference-independent marker of metabolically unhealthy fat distribution. While the comparator model poorly predicts VAT/ASAT ratio (*R*^2^: 0.17–0.26), a silhouette-based model enables significant improvement (*R*^2^: 0.50–0.55). Increased silhouette-predicted VAT/ASAT ratio is associated with increased risk of prevalent and incident type 2 diabetes and coronary artery disease independent of BMI and waist circumference. These results demonstrate that body silhouette images can estimate important measures of fat distribution, laying the scientific foundation for scalable population-based assessment.

## Introduction

Body-mass index (BMI) is a routinely measured proxy for overall fat burden. Increased BMI—used to define obesity in clinical practice—is a leading risk factor for cardiovascular disease, type 2 diabetes, and all-cause mortality^1–4^. While BMI is a useful guide for disease risk at a population level, individuals with the same BMI can have markedly different fat distributions and cardiometabolic risk profiles^5–8^. Prior work utilizing medical imaging such as magnetic resonance imaging (MRI), computed tomography (CT), and dual-energy X-ray absorptiometry (DEXA) has identified certain fat depots as key drivers of “within BMI-group variation” in cardiometabolic risk^9,10^. At any given BMI, increased visceral adipose tissue (VAT) has been associated with cardiometabolic risk while abdominal subcutaneous adipose tissue (ASAT) may have a net neutral effect, and gluteofemoral adipose tissue (GFAT) appears to be a protective “metabolic sink” for excess adipose tissue^11–14^.

These findings suggest potential value in quantifying fat depot volumes—either for identifying high-risk individuals based on metabolically unfavorable characteristics or for tracking response to a given weight reduction therapy. Based on the increased risk conferred by visceral fat, recent professional society guidelines suggest inclusion of waist circumference as a “vital sign” within clinical practice^15^. This is supported by the observation that waist circumference is correlated with VAT volume as well as a strong association of waist circumference with cardiovascular and all-cause mortality^13,16–18^. However, for any given individual, waist circumference may be driven by fat surrounding internal organs (VAT) or fat accumulation just under the skin (ASAT), with potentially important differences in corresponding risk.

Hence, a large gap exists between anthropometric measures such as BMI and waist circumference—easily quantified in clinical practice, but providing limited resolution—and medical imaging, which allows for more precise characterization of fat distribution, but has not been practical to deploy at scale. Images of an individual’s silhouette—if adequately predictive—could close this implementation gap. Prior seminal studies have suggested that estimation of fat distribution using various proxies for medical imaging may be feasible, but have several limitations^19–28^. First, most studies in this area have focused on predicting overall fat and fat-free mass rather than specific fat depots, which is likely to be significantly more diffcult^19–23,28^. Second, no prior study has aimed to predict ratios between fat depots, which are poorly captured by BMI and waist circumference and hence may add the most clinical value^24–27^. Third, prior studies in this area have been limited by sample sizes of up to several hundred healthy participants, limiting the ability to perform robust cross-validation or assess generalizability^19–28^. Fourth, no prior study has demonstrated that fat distribution predicted by an individual’s outline stratifies risk of cardiometabolic disease independent of BMI and waist circumference.

In this study, we derive front- and side-facing silhouette images for 40,032 participants of the UK Biobank from body MRI imaging data. Cross-validated deep learning models trained on these images, using previously calculated whole-body MRI-estimated volumes as truth labels, demonstrate highly accurate estimation of VAT, ASAT, and GFAT volumes, significantly outperforming prediction achievable using anthropometric variables^14^. Beyond measures such as waist circumference, we note that the VAT/ASAT ratio quantified using silhouette images is a strong predictor of both type 2 diabetes and coronary artery disease.

## Results

### Silhouettes allow for accurate estimation of VAT, ASAT, and GFAT volumes

**Fig. 1:**
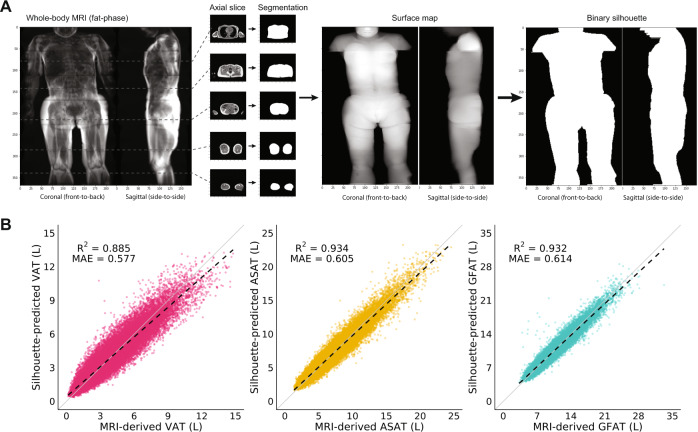
Silhouettes enable estimation of fat depot volumes. **A** Silhouettes were created from body MRIs by segmenting the outline of axial acquisitions, projecting the resulting volume onto a two-dimensional surface map, and binarizing pixels. **B** Silhouette-predicted VAT, ASAT, and GFAT plotted against MRI-derived measurements^14^. Solid black lines denote the linear fits, while the solid gray lines correspond to the identity line. Sex-stratified performance metrics and Bland-Altman plots are available in Supplementary Data 1 and Supplementary Figs. 2–5.

In all, 40,032 participants of the UK Biobank imaging substudy with VAT, ASAT, and GFAT volumes previously quantified on the basis of MRI were included^14,29–32^. Mean age was 65 years, 20,597 (51%) were female, and 97% were white on the basis of self-reported ethnicity (Table 1). Coronal and sagittal silhouettes were generated for each participant by (1) segmenting the body outline in axial MRI acquisitions, (2) computing a surface map of the resulting segmentation volume, (3) projecting this 3-dimensional surface map into 2-dimensional images in the coronal (front-to-back) and sagittal (side-by-side) directions, and (4) converting pixel intensities into binary values, either zeros or ones (Fig. 1A). These silhouettes were used as inputs to train a convolutional neural network (CNN) model to predict VAT, ASAT, and GFAT volumes using MRI-derived measurements as truth labels using a cross-validation procedure (Supplementary Fig. 1)^14^.

**Table 1:** Characteristics of the study population.

	Males (*N* = 19,435)	Females (*N* = 20,597)
Age at time of MRI (years)	65.2 ± 7.7	63.8 ± 7.5
Self-reported ethnicity category		
White	18,773 (96.6)	19,936 (96.8)
Black	137 (0.7)	192 (0.9)
East Asian	112 (0.6)	137 (0.7)
South Asian	238 (1.2)	133 (0.6)
Other	175 (0.9)	199 (1.0)
Blood pressure		
Systolic blood pressure (mmHg)	142.0 ± 17.4	135.8 ± 19.2
Diastolic blood pressure (mmHg)	80.6 ± 9.9	76.7 ± 10.0
Anthropometrics		
Weight (kg)	83.8 ± 13.2	68.9 ± 12.7
Height (cm)	176.3 ± 6.6	162.8 ± 6.35
Body-mass index (kg/m^2^)	27.1 ± 3.8	26.1 ± 4.6
Waist circumference (cm)	94.6 ± 10.5	82.8 ± 11.6
Hip circumference (cm)	100.9 ± 7.3	100.9 ± 9.6
Waist-to-hip ratio	0.94 ± 0.06	0.82 ± 0.07
Fat depot volumes (quantified by MRI)		
Visceral adipose tissue (L)	5.0 ± 2.3	2.6 ± 1.5
Abdominal subcutaneous adipose tissue (L)	5.9 ± 2.5	7.9 ± 3.3
Gluteofemoral adipose tissue (L)	9.3 ± 2.6	11.3 ± 3.2
Cardiometabolic diseases (at time of imaging)		
Type 2 diabetes	1266 (6.5)	638 (3.1)
Coronary artery disease	1545 (7.9)	418 (2.0)
Hypertension	7200 (37.0)	5073 (24.6)
Hypercholesterolemia	5386 (27.7)	3168 (15.4)

Values are reported as means ± SD or as count (percentage).

The CNN trained on silhouettes achieved high performance for predicting VAT (*R*^2^ = 0.885; 95%CI: 0.882–0.887, ASAT (*R*^2^ = 0.934; 0.932–0.935), and GFAT (*R*^2^ = 0.932; 0.930–0.934) volumes (Fig. 1B; Supplementary Data 1; Supplementary Figs. 2–5)^14^. Performance was consistent when the cohort was age-stratified, but attenuated in sex and BMI subgroups, consistent with the significant differences in the distribution of these traits according to sex and BMI (Supplementary Data 1–3; Supplementary Figs. 6 and 7). Performance was broadly consistent across Black, East Asian, and South Asian participants—with the exception of somewhat attenuated VAT prediction in Black participants (*R*^2^ = 0.784; 95%CI: 0.735–0.823)—although these comparisons were limited by lower numbers of non-White individuals (Supplementary Data 4).

### Silhouette-based predictions outperform anthropometric models

We next set out to compare the performance of silhouette-derived predictions of VAT, ASAT, and GFAT volumes with models based on anthropometric measurements. We constructed sex-stratified models combining age with one of—or a combination of—weight, height, body-mass index (BMI), waist circumference, hip circumference, waist-to-hip ratio (WHR), and five bioelectric impedance measurements (Supplementary Data 5).

**Fig. 2:**
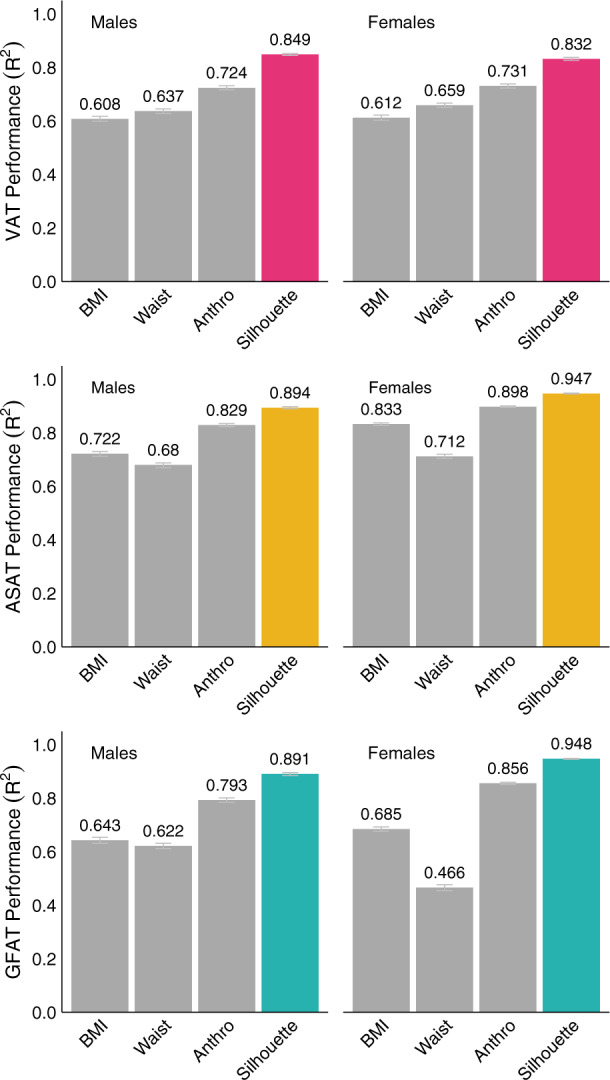
Silhouettes outperform anthropometric models in predicting VAT, ASAT, and GFAT volumes. Sex-specific linear models combining age and various anthropometric metrics measured at the time of imaging were compared to a linear model combining age and silhouette-predicted fat volume (Supplementary Data 5 and 6). Model definitions; BMI: age + BMI; Waist: age + waist circumference; Anthro: age + weight + height + BMI + waist circumference + hip circumference + waist-hip ratio + body impedance measures; Silhouette: age + silhouette. Error bars represent 95% confidence intervals obtained from bootstrapping with 1000 resamples.

BMI-based models offered considerable predictive capacity for each fat depot volume, with the lowest performance observed in male participants for prediction of VAT (*R*^2^ = 0.608; 95% CI: 0.599–0.618) and the best performance observed in female participants for prediction of ASAT (*R*^2^ = 0.833; 95% CI: 0.828–0.837) (Fig. 2 and Supplementary Data 6). These models reflect the high correlation between BMI and any given fat depot volume in the body (Supplementary Fig. 7). Silhouette-based models outperformed BMI-based models by Δ*R*^2^ = 0.220–0.241 for VAT, Δ*R*^2^ = 0.114–0.172 for ASAT, and Δ*R*^2^ = 0.248–0.263 for GFAT, suggesting that significant BMI-independent variation in these three fat depots was captured. In contrast, waist circumference-based models displayed only a small improvement for prediction of VAT (*R*^2^ Male: 0.637; 95% CI: 0.628–0.645; *R*^2^ Female: 0.659; 95% CI: 0.650–0.667) over BMI-based models and performed worse for the prediction of ASAT and GFAT. Finally, we combined all anthropometric and bioimpedance measures in a single model. While improved performance compared to the BMI-based models was observed for VAT (*R*^2^ Male: 0.724; 95% CI: 0.717–0.732; *R*^2^ Female: 0.731; 95% CI: 0.723–0.739), ASAT (*R*^2^ Male: 0.829; 95% CI: 0.823–0.835; *R*^2^ Female: 0.898; 95% CI: 0.895–0.901), and GFAT (*R*^2^ Male: 0.793; 95% CI: 0.785–0.801; *R*^2^ Female: 0.856; 95% CI: 0.852–0.860), silhouette-based models outperformed these models by Δ*R*^2^ = 0.101–0.125 for VAT, Δ*R*^2^ = 0.049–0.065 for ASAT, and Δ*R*^2^ = 0.092–0.098 for GFAT.

We next compared our silhouette-based model for VAT prediction with a recently developed multivariable model for predicting DEXA-derived VAT mass based on 17 anthropometric variables (Supplementary Data 7)^33^. These multivariable models performed similarly to the combined anthropometric model in this study for VAT prediction (*R*^2^ Male: 0.719; 95% CI: 0.709–0.728; *R*^2^ Female: 0.710; 95% CI: 0.694–0.724)—silhouette-based models outperformed these by Δ*R*^2^ = 0.122–0.130.

Taken together, these data suggest that a deep learning model trained on silhouettes can outperform models combining anthropometric and bioimpedance measurements for prediction of VAT, ASAT, and GFAT volumes.

### Silhouette prediction of VAT/ASAT ratio overcomes a key limitation of measured waist circumference

**Fig. 3:**
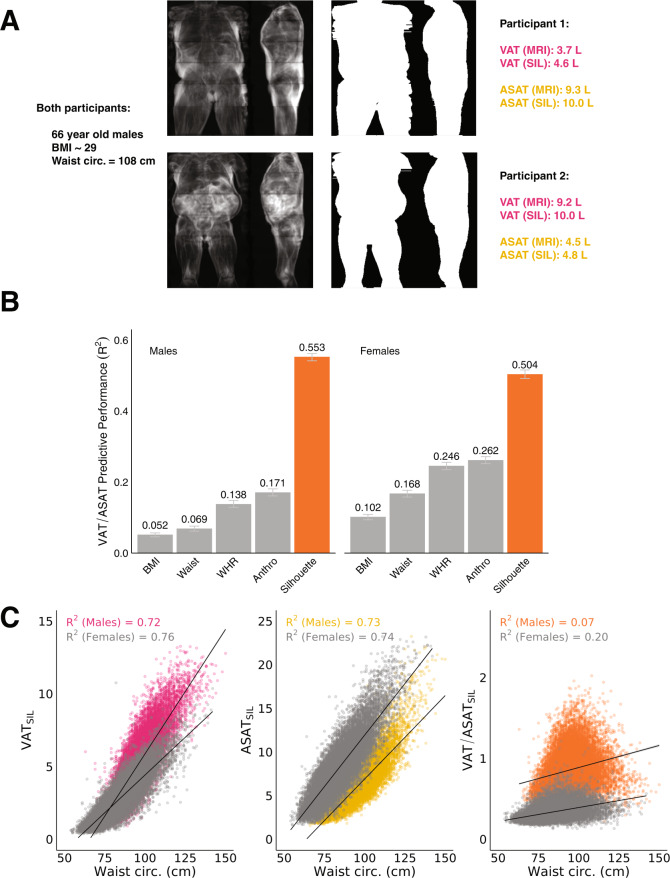
Silhouettes estimate VAT/ASAT ratio, a metric of unfavorable fat distribution. **A** Two-dimensional (2D) MRI projections and silhouettes of an age, sex, BMI, and waist circumference-matched pair of participants with drastic differences in abdominal fat distribution. While both participants have an elevated waist circumference for their sex- and BMI-group, participant 1 primarily has ASAT-driven central obesity, while participant 2 primarily has VAT-driven central obesity. **B** A linear model combining age and silhouette prediction markedly outperforms anthropometric models for the prediction of VAT/ASAT ratio (Supplementary Data 6). Error bars represent 95% confidence intervals obtained from bootstrapping with 1000 resamples. **C** Waist circumference is strongly correlated with silhouette-predicted VAT (VAT_SIL_) and silhouette-predicted ASAT (ASAT_SIL_) (*R*^2^ 0.72–0.76), but nearly independent of silhouette-predicted VAT/ASAT (VAT/ASAT_SIL_) (*R*^2^ 0.07–0.20).

Waist circumference is often used as a proxy for VAT, but the parameter it aims to estimate—central adiposity—can be driven by a preponderence of either ASAT or VAT^15^. As an example, a pair of age, sex, BMI, and waist circumference-matched participants are shown in Fig. 3A with highly discordant abdominal fat distribution—one participant has significantly greater VAT (VAT: 9.2 L, ASAT: 4.5 L, VAT/ASAT ratio = 2.0), while the other has much more ASAT (VAT: 3.7 L, ASAT: 9.3 L, VAT/ASAT ratio = 0.4).

We aimed to investigate the extent to which VAT/ASAT ratio—a marker of metabolically unhealthy fat distribution—could be predicted using anthropometric models. In contrast to their performance for fat depot volumes, sex-specific models combining weight, height, BMI, waist circumference, hip circumference, WHR, and five bioimpendance measures yielded poor predictive performance for VAT/ASAT ratio (*R*^2^ Male: 0.171; 95% CI: 0.161–0.181; *R*^2^ Female: 0.262; 95% CI: 0.252–0.272) (Fig. 3B and Supplementary Data 6). Notably, WHR-based models (without other anthropometric measures) achieved comparable performance (*R*^2^ Male: 0.138; 95% CI: 0.129–0.148; *R*^2^ Female: 0.246; 95% CI: 0.235–0.256).

This marked reduction in performance compared to similar anthropometric models used to predict VAT, ASAT, and GFAT volumes demonstrates the challenge of predicting regional adiposity out of proportion to the overall size of an individual. Much of the predictive performance for fat depot volumes with variables such as BMI and waist circumference comes from an underlying correlation of all of these variables with the overall size of an individual—VAT/ASAT ratio is unique in this regard, being relatively independent of BMI (Pearson *r* Male = 0.14; *r* Female = 0.22) (Supplementary Fig. 7).

We hypothesized that a deep learning model trained on silhouettes could predict VAT/ASAT ratio better than what might be achieved with anthropometric measures, despite the fact that the anatomical boundary between VAT and ASAT cannot be directly ascertained from an individual’s silhouette. Silhouette-based models demonstrated marked improvement over anthropometric models for prediction of VAT/ASAT ratio (*R*^2^ Male: 0.553; 95% CI: 0.542–0.562; *R*^2^ Female: 0.504; 95% CI: 0.492–0.516) (Fig. 3B). Compared to the best anthropometric model, this represented an improvement of Δ*R*^2^ = 0.382 in male participants and Δ*R*^2^ = 0.242 in female participants.

We additionally confirmed that waist circumference was strongly correlated with silhouette-predicted VAT (*R*^2^ Male 0.72; *R*^2^ Female 0.76) and silhouette-predicted ASAT (*R*^2^ Male 0.73; *R*^2^ Female 0.74), but a poor proxy for silhouette-predicted VAT/ASAT (*R*^2^ Male 0.07, *R*^2^ Female 0.20), suggesting that information independent of waist circumference was learned (Fig. 3C and Supplementary Fig. 7).

### Silhouette-predicted VAT/ASAT associates with cardiometabolic diseases

**Fig. 4:**
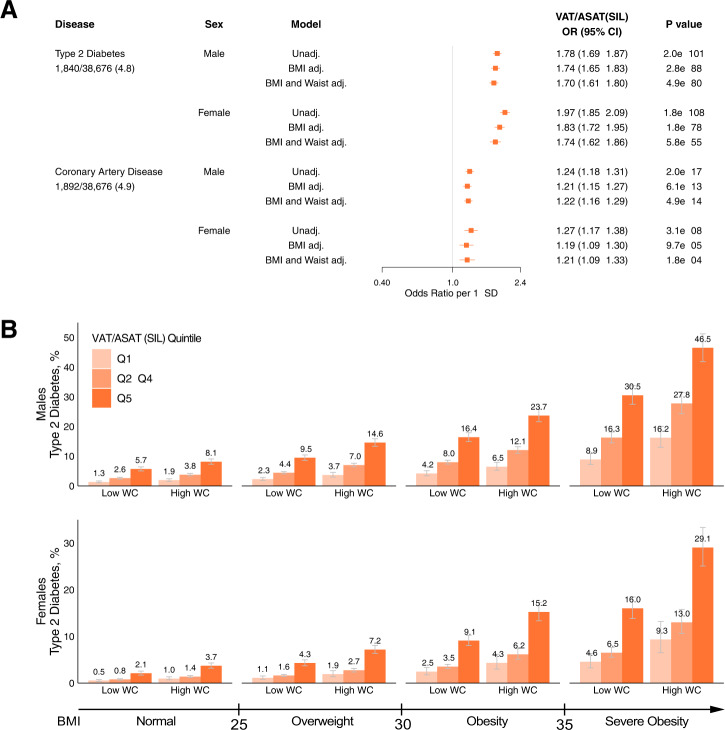
Association of silhouette-predicted VAT/ASAT ratio with type 2 diabetes and coronary artery disease. **A** Disease associations with silhouette-predicted VAT/ASAT ratio in sex-specific unadjusted and adjusted logistic regression models. All models were adjusted for age at the time of imaging and imaging center—“BMI adj” refers to additional adjustment for BMI, while “BMI and Waist adj.” refers to additional adjustment for BMI and waist circumference. Full data are available in Supplementary Data 9. **B** Sex-stratified standardized prevalence of type 2 diabetes across the bottom quintile (light orange), quintiles 2–4 (neutral orange), and the top quintile (dark orange) of silhouette-predicted VAT/ASAT ratio within BMI-bins and waist circumference categories. High waist circumference was defined in a sex- and BMI-subgroup specific fashion as described in Supplementary Data 12. Error bars represent 95% confidence intervals.

We next investigated associations of silhouette-predicted VAT/ASAT with type 2 diabetes, coronary artery disease, hypertension, and hypercholesterolemia (Supplementary Data 8)^34^. In sex-specific logistic regression models adjusted for age and imaging center, silhouette-predicted VAT/ASAT was associated with increased prevalence of type 2 diabetes in both males (OR/SD 1.78; 95% CI: 1.69–1.87) and females (OR/SD 1.97; 95% CI: 1.85–2.09) (Fig. 4A and Supplementary Data 9). Additionally adjusting for BMI and waist circumference minimally attenuated effect sizes for silhouette-predicted VAT/ASAT ratio (OR/SD Male 1.70; 95% CI: 1.61–1.80; OR/SD Female 1.74; 95% CI: 1.62–1.86), consistent with the relative independence of VAT/ASAT ratio with these two anthropometric measures. Similar trends were observed with coronary artery disease, hypertension, and hypercholesterolemia with attenuated effect sizes—for example, in models adjusted for BMI and waist circumference, silhouette-predicted VAT/ASAT associated with increased prevalence of coronary artery disease in both males (OR/SD 1.22; 95% CI: 1.16–1.29) and females (OR/SD 1.21; 95% CI: 1.09–1.33) (Supplementary Fig. 8).

This procedure was repeated with MRI-derived VAT/ASAT in lieu of silhouette-predicted values to compare disease associations. Trends were broadly consistent between MRI-derived VAT/ASAT and silhouette-predicted VAT/ASAT. Interestingly, the association between MRI-derived VAT/ASAT ratio and type 2 diabetes was slightly attenuated compared to silhouette-predicted values in BMI and waist circumference-adjusted models (OR/SD Male 1.43; 95% CI: 1.35–1.51; OR/SD Female 1.50; 95% CI: 1.40–1.62) (Supplementary Fig. 9 and Supplementary Data 10). In contrast, the association with coronary artery disease was nearly identical (OR/SD Male 1.18; 95% CI: 1.12–1.25; OR/SD Female 1.19; 95% CI: 1.08–1.30).

Disease associations with waist-hip ratio (WHR) in place of VAT/ASAT ratio yielded comparable effect sizes, with a somewhat reduced effect size in male participants (Supplementary Fig. 10 and Supplementary Data 11). For example, WHR was associated with increased risk of hypertension in BMI-adjusted analyses (OR/SD Male 1.20; 95% CI: 1.15–1.25; OR/SD Female 1.17; 95% CI: 1.13–1.22) with attenuated effect size compared to silhouette-predicted VAT/ASAT ratio in males (OR/SD Male 1.31; 95% CI: 1.27–1.35; OR/SD Female 1.23; 95% CI: 1.18–1.27). Notably, WHR ratio was only modestly correlated with VAT/ASAT ratio (Pearson r range: 0.35–0.58), suggesting that VAT/ASAT ratio and WHR ratio may independently contribute to disease associations (Supplementary Fig. 7).

We next set out to understand the gradients in disease prevalence rates according to quintiles of silhouette-predicted VAT/ASAT. We estimated prevalence rates for males and females separately across clinical BMI categories of normal, overweight, obese, and severely obese participants with either normal or elevated waist circumference based on previously recommended BMI-specific cutoffs (Supplementary Data 12)^15,35^. This analysis revealed substantial gradients in prevalence of cardiometabolic diseases according to silhouette-predicted VAT/ASAT quintiles, even after stratification within BMI and waist circumference bins (Fig. 4B and Supplementary Data 13). For example, men with overweight BMI and normal waist circumference with silhouette-predicted VAT/ASAT in the top quintile had a higher probability of type 2 diabetes (9.5%; 95% CI 8.6–10.4%) compared to both (1) men with overweight BMI and elevated waist circumference with silhouette-predicted VAT/ASAT in the bottom quintile (3.7%; 95% CI 3.0–4.5%) and (2) men with obese BMI and normal waist circumference with silhouette-predicted VAT/ASAT in the bottom quintile (4.2%; 95% CI 3.4–5.1%). Similar trends were observed for coronary artery disease (Supplementary Fig. 11 and Supplementary Data 13).

Over a median follow-up of 2.8 years after imaging, 235 (0.6%) and 607 (1.5%) participants had a new diagnosis of type 2 diabetes or coronary artery disease recorded in the electronic health record. Silhouette-predicted VAT/ASAT associations with incident disease were broadly consistent with prevalent analyses. In BMI- and waist circumference-adjusted Cox regressions, silhouette-predicted VAT/ASAT associated with increased risk of incident type 2 diabetes (HR/SD Male 1.33; 95% CI: 1.13–1.57; HR/SD Female 1.51; 95% CI: 1.30–1.74) and increased risk of incident coronary artery disease in males (HR/SD 1.19; 95% CI: 1.08–1.30) (Supplementary Data 14). A directionally consistent association was observed with incident coronary artery disease in females, although interpretation was limited by sample size (HR/SD 1.09; 95% CI: 0.94–1.27). Similar effects were observed when MRI-derived VAT/ASAT was used in lieu of silhouette-predicted VAT/ASAT (Supplementary Data 15). Taken together, these data support silhouette-predicted VAT/ASAT ratio as a strong, BMI- and waist circumference-independent predictor of cardiometabolic diseases.

## Discussion

In this study, we developed a deep learning model trained on an individual’s silhouette that predicts VAT, ASAT, GFAT volumes, and VAT/ASAT ratio using cross-validation analyses of 40,032 individuals. These silhouette-based predictions are significantly more accurate than those based on anthropometric and bioimpedance measures, particularly for VAT/ASAT ratio, a metric of unhealthy fat distribution^34^. VAT/ASAT ratio as quantified using silhouette images—largely independent of BMI and waist circumference—was strongly associated with cardiometabolic disease, including diabetes and coronary artery disease. These results have at least three implications.

First, deep learning models trained on simple, less data-rich imaging modalities may help close the gap between sophisticated imaging-based markers of adiposity and clinical impact. The present study is proof-of-concept that input data as simple as the outline of an individual is likely to harbor considerably more information about that individual’s fat distribution compared to models that combine several clinical measurements such as BMI and waist circumference. These silhouette-predicted estimates—while crude in comparison to MRI-derived measurements—may be sufficient for cardiometabolic risk screening. Our findings extend prior work focused on prediction of fat-free mass and related measures from DEXA, to measures of fat distribution trained using more sophisticated MRI-based assessment^19–23^. Recent advances in three-dimensional optical scanners have enabled accurate whole-body surface reconstructions capable of predicting body volumes, waist circumference, and overall fat mass, with some of these products available to the public^22,24,36–38^. Although accurate and non-invasive, the quantities that are most often predicted by these tools are likely to be highly correlated with BMI, waist circumference, and the overall size of an individual. The present study proposes VAT/ASAT ratio as one useful benchmark for determining how much information has been learned about fat distribution independent of BMI. Alongside advances in smartphone camera technology such as LiDAR (light detection and ranging) and recent evidence that smartphone images can quantify overall body fat, our results lay the scientific foundation for a scalable approach to population health management that incorporates assessment of fat distribution^28^.

Second, waist circumference is more strongly correlated with central obesity than BMI, but is unable to distinguish between VAT and ASAT, indices with differing implications for cardiometabolic risk^14,15^. Consistent with this limitation, models incorporating anthropometric measurements did not allow for accurate prediction of VAT/ASAT ratio, which we establish as a largely BMI-independent measure of local adiposity. By contrast, our deep learning model showed good predictive performance for VAT/ASAT ratio, even though the boundary between visceral and subcutaneous fat is not explicitly available in silhouette images. The striking gradients in disease patterns observed for VAT/ASAT ratio were minimally attenuated after additional adjustment for BMI and waist circumference. In light of renewed calls for the routine measurement of waist circumference to better stratify cardiometabolic risk associated with body habitus, our work suggests that VAT/ASAT ratio could provide important additional and largely independent information to inform clinical risk estimation^15^.

Third, the conceptual approach outlined here could be leveraged to identify individuals with undiagnosed lipodystrophies or similar phenotypes currently “flying under the radar” within clinical practice^39^. As an example, familial partial lipodystrophy is a genetic disorder characterized by relative depletion of subcutaneous fat with relative maintenance or excess of visceral fat^40,41^. Prior proof of principle data suggests that it may be possible to differentiate lipodystrophy patients versus controls using a “fat shadow” derived from clinical-grade DEXA imaging^41^. Given that this condition remains under-recognized within practice, systematic assessment of large populations may prove useful in identifying additional individuals who would benefit from genetic testing or a targeted therapy. For example, metreleptin improves the metabolic profile of patients with partial lipodystrophy and tesamorelin selectively reduces visceral fat in patients with HIV despite no impact on BMI and overall weight^42–44^. Beyond monogenic lipodystrophies, there is increasing evidence of a less severe, “polygenic” form of lipodystrophy common in individuals with insulin resistance and less pronounced perturbations in fat distribution^45–47^. Identification of such individuals could, in principle, enable a clinical trial or other assessment of this population to characterize clinical utility.

This study has several limitations, providing opportunities for future investigation. First, the majority of participants in the UK Biobank are white, and the imaged substudy investigated here is of mean age 65 years. Although our data suggests similar performance within participant subgroups based on age and self-reported ethnicity group, additional validation across ancestrally and geographically diverse populations would be of considerable value, especially given prior evidence of significant variability in fat distribution indices across racial groups^48^. An important example relates to the South Asian population, where abnormal fat distribution has been postulated as a key driver of the markedly increased rates of cardiovascular disease and diabetes observed, often in the context of a relatively normal BMI^49,50^. In addition, training on dedicated cohorts with higher BMI is likely to improve predictive accuracy at the extremes of the phenotype distributions. Second, silhouettes in this study were derived by taking the outline of whole-body MRI images, rather than a more cost-effective modality such as photos taken with a smartphone. A future study that utilizes silhouettes obtained from smartphone images would need to additionally account for heterogeneity in user image acquisition technique and require independent validation. Third, we were unable to assess the accuracy of silhouettes in estimating fat depot volume changes over time. Investigation of multiple silhouette-predicted fat depot estimates over time, ideally in the context of a specific lifestyle or clinical intervention, is likely to be of considerable interest.

In conclusion, we demonstrate that a deep learning model using silhouettes can quantify fat distribution phenotypes with important potential clinical implications for cardiometabolic health. These results lay the scientific foundation for a population health effort that allows for tracking of these traits in the general population without the requirement for medical imaging.

## Methods

### Study population

All analyses were conducted in the UK Biobank, a prospective cohort study that recruited over 500,000 individuals aged 40–69 in the UK from 2006 to 2010. In this study, we analyzed 40,032 participants of the imaging substudy with fat depot volumes previously quantified using whole-body MRI^14,29^. This analysis of data from the UK Biobank was approved by the Mass General Brigham Institutional Review Board and was performed under UK Biobank application #7089.

### Preparing silhouettes from whole-body magnetic resonance images

Whole-body MRI data was preprocessed as previously described^14^. In short, whole-body MRIs were acquired in six separate series with varying resolutions, which require preprocessing before merging into three-dimensional (3D) volumes. We resampled each series to the highest available resolution (voxel = 2.232 × 2.232 × 3.0 mm^3^), de-duplicated overlapping regions and merged the six series into 3D volumes. The fat-phase acquisition was used to segment a 3D volume for each individual, as described in the Supplementary Methods.

In order to generate a “silhouette” encoding information only about the outline of an individual, pixel intensities were set to one if they were on the surface of the body and to zero otherwise in either the coronal or sagittal orientation. For example, a given pixel on a coronal two-dimensional projection represents the presence or absence of a segmented pixel in the anterior-posterior direction perpendicular to the coronal plane. Classifying pixels as belonging to either the body or the background, in a procedure known as segmentation, was performed on the two-dimensional axial images. The input to the deep learning models described below was two such silhouette images concatenated side-by-side—one coronal and one sagittal—and resized to 237 × 256 pixels.

### Deep learning to predict fat depot volumes using silhouettes

For predicting the target fat depot volumes, we employed the DenseNet-121 architecture as the base model^51^. Additional information regarding deep learning architecture, parameters, and training procedure can be found in the Supplementary Methods. In brief, we constructed a hierarchical multi-task model with the coronal and sagittal silhouettes as input that jointly predicted VAT, ASAT, and GFAT volumes, and VAT/ASAT ratio **(**Supplementary Methods).

To avoid reporting overfit results and to ensure that all participants received an unbiased prediction, we employed a nested cross-validation approach. In this approach, the cohort is first split into five non-overlapping partitions and five models can then be trained using data from three partitions, then testing and validation is performed using the remaining two partitions (Supplementary Fig. 1). For each model, predictions from the validation partition are unbiased and are collected to acquire predictions for all participants. We found that performing cross-validation within the partitions improved performance. The final prediction for each fold was reported as the mean-ensemble of the cross-validation models.

### Linear anthropometric models to benchmark performance

Sex-specific anthropometric models were generated by predicting each MRI-derived fat measurement using one of, or a combination of, age, weight, height, body-mass index (BMI), waist circumference, hip circumference, waist-to-hip ratio (WHR), and five bioelectric impedance measurements commonly used for measuring body fat. We utilized the aforementioned nested cross-validation approach to generate predictions from these models. *R*^2^ and mean absolute error (MAE) are reported to compare performance of models. 95% confidence intervals for *R*^2^ were generated by bootstrapping with 1000 resamples.

### Association with cardiometabolic diseases

The primary outcomes were prevalent and incident type 2 diabetes and coronary artery disease, and prevalent hypertension and hypercholesterolemia^14^. Type 2 diabetes was defined on the basis of ICD-10 codes, self-report during a verbal interview with a trained nurse, use of diabetes medication, or a hemoglobin A1C greater than 6.5% before the date of imaging (Supplementary Data 8). Coronary artery disease was defined as myocardial infarction, angina, revascularization (percutaneous coronary intervention and/or coronary artery bypass grafting), or death from CAD as determined on the basis of ICD-10 codes, ICD-9 codes, OPCS-4 surgical codes, nurse interview, and national death registries. Hypertension was defined on the basis of ICD-10 codes, ICD-9 codes, nurse interview, or diagnosis by a doctor, and hypercholesterolemia was defined on the basis of ICD-10 codes or nurse interview, as previously described^52^.

Sex-stratified logistic regression models adjusted for age at time of imaging, imaging center, BMI, and waist circumference were used to test associations of silhouette-predicted VAT/ASAT ratio with prevalent disease. Cox proportional-hazard models with the same covariates were used to test associations of silhouette-predicted VAT/ASAT ratio with incident events. Finally, we used sex-stratified logistic regression models adjusted for the same covariates to determine the gradient in probability of prevalent disease across quintiles of silhouette-predicted VAT/ASAT ratio in BMI- and waist circumference-bins.

### Reporting summary

Further information on research design is available in the Nature Research Reporting Summary linked to this article.

## Supplementary information

Supplementary Information

Supplementary Data 1-16

Reporting Summary

## Data Availability

The raw UK Biobank data—including the anthropometric data reported here—are made available to researchers from universities and other research institutions with genuine research inquiries following IRB and UK Biobank approval.

## Code availability

Representative code used in this work will be deposited at the following Github repository: https://github.com/broadinstitute/ml4h/tree/master/model_zoo/silhouette_mri.
