# Supplementary material for: Silhouette images enable estimation of body fat distribution and associated cardiometabolic risk
Source: NPJ Digit Med. 2022 Jul 27;5:105. doi: 10.1038/s41746-022-00654-1 (PMC9329470; doi:10.1038/s41746-022-00654-1)
Supplement: Supplementary file 3 — Reporting Summary [file 41746_2022_654_MOESM3_ESM.pdf]

nature portfolio

Corresponding author(s): Marcus D.R. Klarqvist, Saaket Agrawal, and Amit V. Khera

Last updated by author(s): Jul 4, 2022

Reporting Summary

Nature Portfolio wishes to improve the reproducibility of the work that we publish. This form provides structure for consistency and transparency in reporting. For further information on Nature Portfolio policies, see our Editorial Policies and the Editorial Policy Checklist.

Statistics

For all statistical analyses, confirm that the following items are present in the figure legend, table legend, main text, or Methods section.

n/a	Confirmed	
☐	☒	The exact sample size (*n*) for each experimental group/condition, given as a discrete number and unit of measurement
☐	☒	A statement on whether measurements were taken from distinct samples or whether the same sample was measured repeatedly
☐	☒	The statistical test(s) used AND whether they are one- or two-sided<br>*Only common tests should be described solely by name; describe more complex techniques in the Methods section.*
☐	☒	A description of all covariates tested
☐	☒	A description of any assumptions or corrections, such as tests of normality and adjustment for multiple comparisons
☐	☒	A full description of the statistical parameters including central tendency (e.g. means) or other basic estimates (e.g. regression coefficient) AND variation (e.g. standard deviation) or associated estimates of uncertainty (e.g. confidence intervals)
☐	☒	For null hypothesis testing, the test statistic (e.g. *F*, *t*, *r*) with confidence intervals, effect sizes, degrees of freedom and *P* value noted<br>*Give P values as exact values whenever suitable.*
☒	☐	For Bayesian analysis, information on the choice of priors and Markov chain Monte Carlo settings
☒	☐	For hierarchical and complex designs, identification of the appropriate level for tests and full reporting of outcomes
☐	☒	Estimates of effect sizes (e.g. Cohen's *d*, Pearson's *r*), indicating how they were calculated

Our web collection on statistics for biologists contains articles on many of the points above.

Software and code

Policy information about availability of computer code

Data collection	Whole-body magnetic resonance imaging data was downloaded from the UK Biobank showcase (https://biobank.ndph.ox.ac.uk/showcase/)
Data analysis	Code used to ingest silhouettes from whole-body Dixon MRIs and trained deep learning models are available at https://github.com/broadinstitute/ml4h/tree/master/model_zoo/silhouette_mri under an open-source BSD license.

For manuscripts utilizing custom algorithms or software that are central to the research but not yet described in published literature, software must be made available to editors and reviewers. We strongly encourage code deposition in a community repository (e.g. GitHub). See the Nature Portfolio guidelines for submitting code & software for further information.

Data

Policy information about availability of data

All manuscripts must include a data availability statement. This statement should provide the following information, where applicable:

- Accession codes, unique identifiers, or web links for publicly available datasets
- A description of any restrictions on data availability
- For clinical datasets or third party data, please ensure that the statement adheres to our policy

The raw UK Biobank data - including the anthropometric data reported here - are made available to researchers from universities and other research institutions with genuine research inquiries following IRB and UK Biobank approval. Representative code used in this work will be deposited at the following Github repository: https://github.com/broadinstitute/ml4h/tree/master/model_zoo/silhouette_mri.

Field-specific reporting

Please select the one below that is the best fit for your research. If you are not sure, read the appropriate sections before making your selection.

☒ Life sciences ☐ Behavioural & social sciences ☐ Ecological, evolutionary & environmental sciences

For a reference copy of the document with all sections, see nature.com/documents/nr-reporting-summary-flat.pdf

Life sciences study design

All studies must disclose on these points even when the disclosure is negative.

Sample size	A total of 40,032 whole-body MRIs were used to derive whole-body binary silhouettes
Data exclusions	Participants that failed MRI imaging QC, including parts of the image missing, fat/water swaps, and imaging artifact in the liver, were excluded leading to 40,032 participants available for analysis in this study. No additional exclusions were made.
Replication	All deep-learning models results were replicated by a nested cross-validation approach.
Randomization	For cross-validation, participants were randomly allocated to one of each k-fold group for training, testing, and validating models.
Blinding	The investigators were blind to participants characteristics as they were randomly allocated to one of each k-fold group for training, testing, and validating models.

Reporting for specific materials, systems and methods

We require information from authors about some types of materials, experimental systems and methods used in many studies. Here, indicate whether each material, system or method listed is relevant to your study. If you are not sure if a list item applies to your research, read the appropriate section before selecting a response.

Materials & experimental systems

n/a	Involved in the study	
☒	☐	Antibodies
☒	☐	Eukaryotic cell lines
☒	☐	Palaeontology and archaeology
☒	☐	Animals and other organisms
☐	☒	Human research participants
☒	☐	Clinical data
☒	☐	Dual use research of concern

Methods

n/a	Involved in the study	
☒	☐	ChIP-seq
☒	☐	Flow cytometry
☒	☐	MRI-based neuroimaging

Human research participants

Policy information about studies involving human research participants

Population characteristics	All analyses were conducted in the UK Biobank, a richly phenotyped, prospective, population-based cohort that recruited over 500,000 individuals aged 40-69 in the UK from 2006 to 2010.
Recruitment	The UK Biobank comprises of 500,000 individuals aged 40-69 in the UK and were recruited via mailer from 2006-2010
Ethics oversight	This analysis of data from the UK Biobank was approved by the Mass General Brigham Institutional Review Board and was performed under UK Biobank application #7089

Note that full information on the approval of the study protocol must also be provided in the manuscript.
